# Antioxidant Properties of *Artemisia annua* Extracts in Model Food Emulsions

**DOI:** 10.3390/antiox3010116

**Published:** 2014-03-03

**Authors:** Monika Skowyra, Maria Gabriela Gallego, Francisco Segovia, Maria Pilar Almajano

**Affiliations:** Chemical Engineering Department, Technical University of Catalonia, Av. Diagonal, 649, Barcelona 08034, Spain; E-Mails: skowyra.monika@gmail.com (M.S.); maria.gabriela.gallego@upc.edu (M.G.G.); segoviafj@gmail.com (F.S.)

**Keywords:** *Artemisia annua*, antioxidants, oil-in-water emulsions, lipid oxidation

## Abstract

*Artemisia annua* is currently the only commercial source of the sesquiterpene lactone artemisinin. Although artemisinin is a major bioactive component present in this Chinese herb, leaf flavonoids have shown a variety of biological activities. The polyphenolic profile of extract from leaves of *A. annua* was assessed as a source of natural antioxidants. Total phenolic content and total flavonoid content were established and three assays were used to measure the antioxidant capacity of the plant extract. The measurement of scavenging capacity against the 2,2′-azino-bis-3-ethylbenzothiazoline-6-sulphonic acid (ABTS) radical cation, the oxygen radical absorbance capacity (ORAC) and the ferric reducing antioxidant power (FRAP) were 314.99 µM Trolox equivalents (TE)/g DW, 736.26 µM TE/g DW and 212.18 µM TE/g DW, respectively. *A. annua* extracts also showed good antioxidant properties in 10% sunflower oil-in-water emulsions during prolonged storage (45 days) at 32 °C. Artemisia extract at 2 g/L was as effective as butylated hydroxyanisole (BHA) at 0.02 g/L in slowing down the formation of hydroperoxides as measured by peroxide value and thiobarbituric acid reactive substances. The results of this study indicate that extract of *A. annua* may be suitable for use in the food matrix as substitutes for synthetic antioxidants.

## 1. Introduction

Lipid oxidation is of great concern to the consumer because it causes physical and chemical deterioration of food quality, such as undesirable changes in taste, texture, appearance and development of rancidity, losses of important nutritional values and formation of potentially harmful components including free radicals and reactive aldehydes [1,2]. Especially, this process is favored in oil-in-water emulsions because of the large contact surface between the oxidizable lipid hydroperoxides in emulsion droplets and water-soluble prooxidants resulting in the propagation of oxidation reactions [3]. To avoid this problem, synthetic antioxidants are commonly used, such as butylated hydroxytoluene and butylated hydroxyanisole [4]. However, in recent years there has been an increasing interest in the use of naturally occurring substances for the preservation of food. Aromatic plants have been the subject of study, particularly by the chemical, pharmaceutical and food industries, because of their potential use in food for two principal reasons: (i) safety considerations regarding the potentially harmful effects of the chronic consumption of synthetic compounds in food and beverages; and (ii) “natural” additives are perceived as beneficial for both quality and safety aspects and also possible beneficial effects on human health [5]. 

*Artemisia annua* (*Asteraceae* family) commonly known as “annual wormwood” is a plant used for many centuries in Chinese folk medicine for the treatment of malaria and fever. Its health-promoting effects have been mainly attributed to its content of artemisinin, a sesquiterpene lactone used as the raw material for production of artemisinin-based combination therapy, used against drug-resistant *Plasmodium falciparum* in areas where malaria is endemic. *A. annua* is also a rich source of antioxidant flavonoids that are thought to play an important role in potentiating the effects of artemisnin drugs against cancer and parasitic diseases [6]. Moreover, *A. annua* leaves have a high content of essential oil (EO) containing cineole, α-pinene, camphene, camphor and artemisia ketone [7]. The essential oil of *A. annua* is referenced as having antifungal and antimicrobial activity [8]. *A. annua* also shows anti-inflammatory, antipyretic [9], antioxidant [10], anticancer [11,12] and cytotoxic [13] activities. Although not yet reported in the literature, *A. annua* extracts, being a rich source of various phenolic compounds could therefore be incorporated in model emulsions as a source of natural antioxidant to prolong quality and stability.

The aim of this paper is to report a study of the antioxidant properties of *Artemisia annua* extracts in model emulsions stored for long periods, which can be representative of real food systems and their expected shelf life. Lipid oxidation was determined by following the formation of peroxide values (PV) as the primary oxidation products and thiobarbituric acid reactive substances (TBARs) as the secondary products.

## 2. Experimental Section

### 2.1. Materials

*Artemisia annua* was grown in a greenhouse (Balaguer, Spain). Leaves of *A. annua* were collected, dried and ground to a homogenous powder in collaboration with the company Pàmies Hortícoles. Refined sunflower oil was purchased in a local market. All reagents and chemicals were of analytical grade supplied by Sigma–Aldrich Company Ltd. (Gillingham, UK) or Panreac (Barcelona, Spain).

### 2.2. Extraction

Air-dried and finely ground *Artemisia annua* was weighed (2 g) and extracted with 50 mL of ethanol-water mixture at 50:50 (v/v). The mixture was stirred continuously for 24 h at 4 °C. After that, all samples were centrifuged (Sigma 6K10, Osterode am Harz, Germany). Part of the supernatant was used to determine the antiradical capacity. The volume of the remaining supernatant was measured and the solution was evaporated, frozen at −80 °C for 24 h and lyophilized for 3 days. Samples were then weighed and kept protected from light in a desiccator until used to prepare an oil-in-water emulsion system.

### 2.3. Total Phenol and Flavonoid Content

Total polyphenol content (TPC) of extracts was determined by colorimetry following the Folin-Ciocalteu method [14]. The absorbance was measured at 725 nm using a UV–vis spectrophotometer (Fluostrar Omega, Perkin-Elmer, Paris, France) and the results were expressed in gallic acid equivalents, GAE, using a gallic acid standard curve (10–70 µM).

Total flavonoid content (TFC) of extracts was measured according to the method of Zhishen *et al*. [15]. The absorbance at 510 nm was measured using spectrophotometer UV-4201/20 (Zuzi, AuxiLab, S.L., Navarra, Spain). Values were determined from a calibration curve prepared with catechin (ranging from 6 to 60 mg/L) and expressed as mg of catechin equivalent per gram of dry weight of plant (CE/g DW).

### 2.4. Antioxidant Capacity Determination

Three different methods were used for the evaluation of the antioxidant activity of the extracts: 2,2′-azino-bis-(3-ethylbenzthiazoline)-6-sulphonic acid (ABTS^●+^) assay [16], Oxygen Radical Absorbance Capacity (ORAC) assay [17] and Ferric Reducing Antioxidant Power (FRAP) method [18]. Results were expressed as µM of Trolox equivalent (TE) per gram of dry weight of plant (DW).

### 2.5. Liquid Chromatography-Mass Spectrometry

LC-MS analyses of the *A. annua* extracts were carried out using LC-QTOF-MS instrument, acquired from Agilent (Wilmington, DE, USA). The LC was an Agilent 1200 Series, consisting of a vacuum degasser unit, an autosampler, two isocratic high pressure mixing pumps and a chromatographic oven. The QTOF mass spectrometer was an Agilent 6520 model, furnished with a Dual-Spray ESI source. The mobile phase was composed of 0.1% formic acid (v/v) in water (eluent A) and 0.5% formic acid (v/v) in acetonitrile (eluent B). Separations were performed on a reversed-phase Zorbax Eclipse XDB-C18 column (100 mm × 2.1 mm, 3.5 µm) acquired from Agilent and connected to a C18 (4 mm × 2 mm) guard cartridge supplied by Phenomenex (Torrance, CA, USA). The temperature of the column was maintained at 30 °C, the mobile phase flow was 0.2 mL/min, and the following gradient was used: 0–10 min, 3% B; 10–25 min, 100% B; 27–38 min, 3% B. The injection volume for samples was 10 µL. Nitrogen (99.999%), used as nebulizing (35 psi) and drying gas (330 °C, 10 °C/min) in the dual ESI source, was provided by a high purity generator (ErreDue srl, Livorno, Italy). Nitrogen (99.9995%), for collision-induced dissociation experiments (MS/MS measurements), was purchased from Carburos Metálicos (A Coruña, Spain). The QTOF instrument was operated in the 2 GHz (Extended Dynamic Range, mass resolution from 4500, at *m/z* 100, to 11,000, at *m/z* 900) mode and compounds were ionized in positive ESI, applying capillary and fragmentor voltages of 3500 and 160 V, respectively. A reference calibration solution (Agilent calibration solution A) was continuously sprayed in the source of the QTOF system, through a second nebulizer. The Mass Hunter Workstation software was used to control all the acquisition parameters of the LC-ESI-QTOF-MS system and also to process the obtained data. Full scan MS spectra were acquired in the range from 100 to 1700 *m/z* units, during the whole chromatographic run, considering an acquisition rate of 1.4 spectra/s. The identification (caffeic acid, apigenin and rutin) was based on the accurate masses, isotopic abundances and spacing of signals in their ([M + H]^+^) cluster of ions, obtained in the MS mode, as well as, on their MS/MS fragmentation patterns and the exact mass of products ions.

### 2.6. Oil-in-Water Emulsion System

#### 2.6.1. Removal of Tocopherols from Sunflower oil

Tocopherols were removed from sunflower oil by column chromatography using activated alumina, as described by Yoshida *et al*. [19]. The oil was stored at −80 °C prior to emulsion preparation (up to 2 days).

#### 2.6.2. Preparation of Emulsions and Storage Conditions

Oil-in-water emulsions were prepared with 1% of Tween 20 as emulsifier and 10% of sunflower oil (2.7.1). Emulsions were prepared by dropwise addition of oil to the water phase, with sonication using a UP200S ultrasonic (Hielscher Ultrasonics GmbH, Teltow, Germany) while cooling in an ice bath for 10 min. It was necessary to repeat sonication 7 times (7 × 10 min) to have enough volume of emulsion. Freeze-dried powder of the *A. annua* extract was redissolved in ethanol 50% (v/v) and added directly to the emulsion and homogenized, obtaining final concentrations of 0.20, 0.65 and 2 g/L (C1, C2 and C3, respectively). For the negative control no extract was added, and the positive controls were prepared with Trolox (0.02 g/L) and BHA (0.02 g/L) dissolved in ethanol.

All emulsions were stored in triplicate in 30 mL amber bottles in the dark, with constant elliptical movement and allowed to oxidize at 32 ± 1 °C for 45 days.

#### 2.6.3. Measurement of Primary Oxidation by Peroxide Value (PV) and pH

Peroxide value (PV) was measured periodically (every 2 or 3 days during the time of storage) using aliquots of 0.007–0.01 g of each sample and determined by the ferric thiocyanate method [20], after calibrating the procedure with a series of oxidized oil samples analyzed by the AOCS Official Method Cd 8-53 [21].

The pH of the samples was measured (pH-meter GLP21, Criston Instruments, Barcelona, Spain) as a parameter to investigate its correlation with PV.

#### 2.6.4. Measurement of Secondary Oxidation by TBARs Method

The thiobarbituric acid reactive substances (TBARs) assay was performed as described by Maqsood and Benjakul [22] with some modifications. One mililiter of oil-in-water emulsion sample was mixed with a TBARs solution containing 0.375% thiobarbituric acid and 15% trichloroacetic acid in 0.25 N HCl solution (5 mL). The samples were placed immediately in an ultrasonic bath (Prolabo brand equipment, Lutterworth, UK) for 5 min and then heated in a water bath (95 °C) for 10 min. The mixture was centrifuged (Sigma 3K30, Sigma Laborzentifugen GmbH, Osterode am Harz, Germany) at room temperature at 4000 rpm for 10 min. The absorbance of the supernatants was measured at 532 nm (Spectrophotomter UV-4201/20, Zuzi, Navarra, Spain). The TBARs values were expressed as mg of malondialdehyde (MDA) per kg of emulsion calculated using 1,1,3,3-tetraethoxypropane (Sigma-Aldrich, St. Louis, MO, USA) as the standard.

### 2.7. Statistical Analysis

TPC, TFC, ABTS^+^, ORAC and FRAP measurements were performed in triplicate on triplicate samples. PV and TBARs measurements were performed once on triplicate samples. 

Mean values for different parameters were calculated and compared by analysis of variance (one-way ANOVA) using commercial software (Minitab 16). Moreover, statistical differences between mean values were identified at the 95% of confidence level (*p* < 0.05). Person’s correlation analysis was performed using the same statistical package.

## 3. Results and Discussion

### 3.1.Phenolic Content and *in-Vitro* Antioxidant Activity of Extract

The total polyphenols (TPC) and flavonoids (TFC) in extracts of *A. annua* leaves obtained with 50% ethanol are shown in Table 1. The *A. annua* extract contained 23.36 ± 0.92 mg gallic acid (GAE)/g dry weight (DW) and 2.68 ± 0.07 mg catechin/g DW (TPC and TFC, respectively). 

**Table 1:** Polyphenol and flavonoid content and antioxidant activity of *A. annua* extracts.

Method	Amount detected *
Total polyphenol content (mg GAE/g DW)	23.36 ± 0.92
Total flavonoid content (mg CE/g DW)	2.68 ± 0.07
ABTS (µM TE/g DW)	314.99 ± 7.70
ORAC (µM TE/g DW)	736.26 ± 17.55
FRAP (µM TE/g DW)	212.18 ± 6.02

* Results are expressed as mean ± standard deviation (*n* = 3).

A recent paper on the analysis of extracts of *A. annua* [23] found a TPC values (384.1 ± 6.7 to 521.2 ± 5.4 mg GAE/100 g DW) for methanol and acetone extraction, respectively, much lower than what we report here for ethanolic extract. However, studies involving hexane and methanol extraction of *A. annua* leaves have reported higher values than those obtained in the present study, in the range of 90.12–134.50 mg GAE/g DW [24]. In addition the same authors found higher TFC value (6.14 mg epicatechin/g DW) in the methanolic extract. Consequently, the extraction method and the solvent used play a key role in the extraction of polyphenols and flavonoids from plant material. 

Antioxidant activity of the extracts from *A. annua* was assessed by three different methods: ABTS, ORAC and FRAP. The use of several methods provides more comprehensive information about the antioxidant properties of the original product because there are substantial differences in sample preparation, extraction of antioxidants (solvent, temperature, *etc*.), selection of end-points and expression of results [5]. For the ABTS assay the value obtained was 314.99 ± 7.70 µM TE/g DW, a value 2 times lower than that found in the ORAC assay which was 736.26 ± 17.55 µM TE/g DW. It is quite usual to obtain higher values in the ORAC test, due to differences in the sensitivity of these methods. Finally, for the FRAP assay the value found was 212.18 ± 6.02 µM TE/g DW. Gouveia and Castillo [23] found the ABTS value of 477.0–2197.3 µM TE/100 g DW in *A. annua* leaves using extraction with methanol and acetone, respectively, which is much lower than that we found in the current study. Also Zheng and Wang [25] found the ORAC value (15.69 ± 0.57 µM TE/g fresh weight) in the phosphate buffer extract much lower than what we report here for the alcoholic extract. Viuda-Martos [5] described the ferric reducing capacity and metal chelating ability of the *A. annua*, finding strong reducing power and effectivity in metal chelating (62.25%–98.03%) of essential oils from *A. annua*. They also reported determination of oxidative stability of fat (Rancimat assay), finding that 5–50 g/L *A. annua* essencial oils showed pro-oxidant activity.

A few recent reports indicated that *A. annua* was one of the four medicinal plants with the highest ORAC level, the ORAC value of *A. annua* leaves and inflorescences extracts was reported as 1125 and 1234 µM TE/g, respectively, which is half to two thirds of the ORAC of oregano extracts [6].

LC-MS analysis of the plant extract of *A. annua* showed the presence of several phenolic compounds quantified in the following increasing order: caffeic acid, rutin and apigenin (Table 2). The concentrations of caffeic acid (1.352 µg/g DW), rutin (0.765 µg/g DW) and apigenin (0.135 µg/g DW) in *A.annua* extract were lower than those reported in the literature. Carvalho *et al*. [26] reported that the *A. annua* leaves conteined 80 µg/g of DW of catechins, 2 µg/g of DW of flavonols, 75 µg/g of DW of hydroxycinnamic acids and 430 µg/g of DW of hydroxybenzoic acids. Carbonara *et al*. [27] found in water extracts of *A. annua* 3.11 ± 0.02–4.10 ± 0.06 mg/g DW of caffeic acid. Morover, Ivanescu *et al*. [28] reported that *A. annua* had 1.144 mg/100 g DW of apigenin.

**Table 2:** Liquid chromatography-mass spectrometry (LC-MS) parameters and amount of selected antioxidant compounds in *A. annua* extracts.

Compounds	Rt (min)	Linear regression equation	*R*^2^	Linear range (ppm)	MS (*m/z*) [M − H]	Content µg/g DW
Rutin	5.33	*y* = 333.54*x* + 2184.6	0.998	0.1–1	609	0.764
Caffeic acid	5.41	*y* = 588.03*x* + 198.38	0.999	0.1–1.5	179	1.353
Apigenin	7.85	*y* = 1028.4*x* + 37085	0.991	0.1–0.5	269	0.135

### 3.2. Antioxidant Activity of Extracts in Model Emulsion System

In this study, to accelerate the oxidative damage, emulsions were stored at 32 ± 1 °C. Oxidative stability was assessed by periodic analysis of primary and secondary oxidation products (measured by the peroxide and the thiobarbituric acid reactive substances values, respectively). In addition the change in pH was monitored, since pH tends to fall during oxidation. 

**Figure 1:**
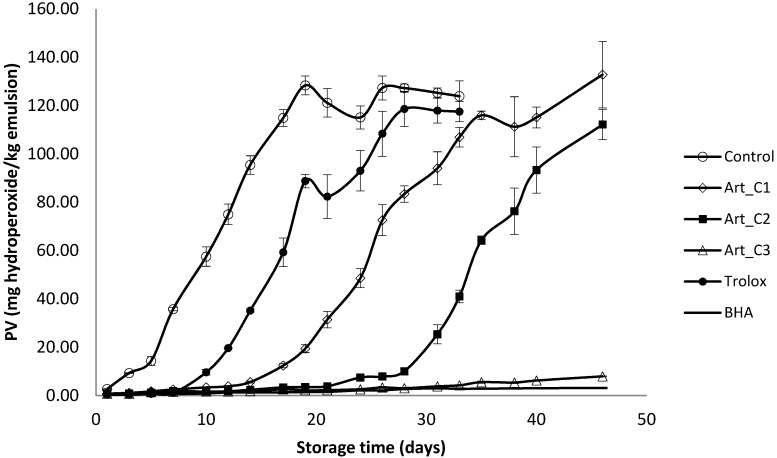
Evaluation of primary oxidation (peroxide value) in a model food system (O/W emulsion 10% of oil) with different concentrations of *A. annua* (C1: 0.20 g/L; C2: 0.65 g/L and C3: 2 g/L).

Peroxide values in the oil emulsions increased significantly faster in the sample without any antioxidant addition (Figure 1), reaching 10 meq hydroperoxides/kg of emulsion (this value is the allowed limit for products containing edible fats) after four days. The next samples to reach this level of deterioration were Trolox at 0.02 g/L (after 10 days), Art_C1 at 0.20 g/L (after 16 days) and Art_C2 at 0.65 g/L (after 28 days). Other samples: Art_C3 and BHA were stable until the end of the experiment (after 45 days, PV was <10 meq/kg). *A. annua* extracts added to oil-in-water emulsions were very effective in stablilzing the emulsion with 2 g/L *A. annua* extract being similar to BHA (0.02 g/L) in activity during 45 days of storage at 32 °C. Kiokias *et al*. [29] reported peroxide values between 45.60 and 51.15 meq/kg after two months in 10% sunflower oil-in-water emulsions with 2 g/L of different carotenoids including β-carotene, lycopene, paprika, lutein and bixin. Ramful *et al*. [30] found that *Eugenia pollicina* leaf extract at a concentration of 0.02% was also effective in slowing down hydroperoxide formation in soybean oil emulsion during 13 days of storage at 40 °C. Roedig-Penman *et al*. [31] reported that tea extracts added to sunflower oil-in-water emulsion were very effective in its stabilization, the tea extract (0.03%) being similar to BHT (0.02%) and taking 40 days of storage at 30 °C to reach a PV of 30 meq/kg.

**Figure 2:**
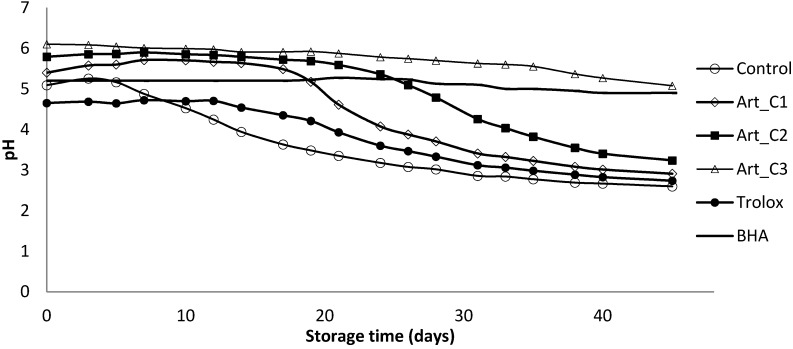
Evaluation of pH in a model food system (O/W emulsion 10% of oil) with different concentrations of *A. annua* (C1: 0.20 g/L; C2: 0.65 g/L and C3: 2 g/L).

pH can affect oxidative reactions by influencing prooxidant (e.g., iron solubility increases with decreasing pH) and antioxidant (the pH can alter the charge of antioxidants, which can affect solubility and chelation capacity) activity. The pH of oxidation models should therefore be similar to the food of interest [32]. In addition, since it is known that many antioxidant molecules are less effective when the pH is low [33], this parameter was also measured as a potential indicator of oil-in-water emulsions oxidation. From an initial average value of 5.5, the samples without any antioxidant addition and with Trolox tended to stabilize their pH at 2.60 and 2.74, respectively, after 45 days (Figure 2). In the Art_C1, Art_C2, Art_C3 and BHA samples the pH slowly decreased during storage, but in Art_C1 and Art_C2 it decreased rapidly after 25 and 33 days, reaching the value of 2.90 and 3.24, respectively. Observing this relationship confirmed that the pH fell as PV increased. Gallego *et al*. [18] and Sorensen *et al*. [34] reported that lipid oxidation increased when pH was decreased from 6 to 3 in a 10% oil-in-water emulsion. 

**Figure 3:**
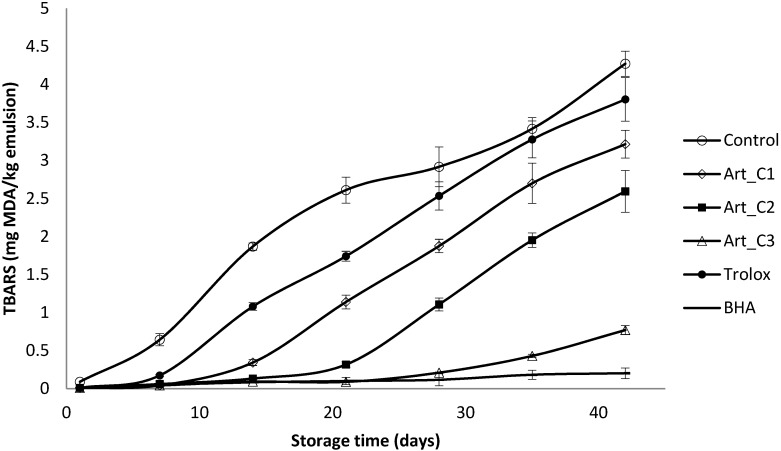
Evaluation of secondary oxidation (TBARs) in a model food system (O/W emulsion 10% of oil) with different concentration of *A. annua* (C1: 0.20 g/L; C2: 0.65 g/L and C3: 2 g/L).

Secondary oxidation products in the emulsions were monitored by measurement of the TBARs (Figure 3). After 6 weeks, TBARs values in emulsions containing *A. annua* extracts and BHA were lower than that those in the control (4.27 mg MDA/kg) and the Trolox-containing sample (3.80 mg MDA/kg). BHA was the most effective antioxidant followed by *A. annua* extract Art_C3, Art_C2 and Art_C1. Garcia-Iñiguez *et al*. [35] reported that a lyophilized aqueous extract of *Melissa officinalis* (lemon balm) at 620.6 ppm was as efficient as BHA at 200 ppm in controlling the TBARs formation in oil-in-water emulsions made with a mixture of algae and lineseed oils upon storage during 15 days at 20 °C. Dimakou and Oreopoulou [36] found that polar (paprika, marigold, bixin) and hydrophobic (β-carotene, lycopene) carotenoids exerted antioxidant effect measured by TBARs test during thermally accelerated autooxidation (60 °C) of sunflower oil-in-water emulsions stabilized by Tween 20. 

In the present study positive correlation between PV and TBARs (*R*^2^ = 0.9200) levels in oil-in-water emulsions was found.

The activity of phenolic compounds as antioxidants in food systems (such as oil-in-water emulsions) depends not only on the structure (*i.e*., number and position of hydroxyl groups bound to the aromatic ring) and chemical reactivity of the phenolics but also on other factors such as their physical location, interactions with other food components, and environmental conditions, for example pH [2,34,37]. Natural plant antioxidants can protect food components from oxidation under the stress of heating and storage. The most effective antioxidants are those that interrupt the free radical chain reaction. Usually containing aromatic or phenolic rings, these antioxidants donate H^•^ to the free radicals formed during oxidation becoming radicals themselves. These radical intermediates are stabilized by the resonance delocalization of the electron within the aromatic ring and formation of quinone structures. In addition, in many of the phenolics positions suitable for molecular oxygen attack are not available. Both synthetic (BHA and BHT) and natural plant antioxidants contain phenolic (flavonoid) functions. Plant extracts with antioxidant activity generally quench free radical oxygen with phenolic compounds as well [4]. However, the addition of polyphenols to lipid dispersions has been shown to result not only in antioxidant effects [38], but also in pro-oxidant activity [39]. 

Phenolic compounds such as caffeic acid, rutin and apigenin have received increasing interest due to their potential antioxidant activity. Caffeic acid has a single aromatic ring with two –OH groups that are capable of donating H^•^. In addition it is a polar compound with a strong ability for chelating metals [4]. Rutin is a compound that contains an *o*-diphenol group in their molecular structure (*o*-diphenol groups are able to chelate metal ions such as iron) [34].

The antioxidant capacity of natural extracts in food emulsions bas been ascribed to a number of influential factors, including the different polarities and antiradical activities of mixed phenolics. The presence of water in the emulsion results in the partition of antioxidants between polar and apolar phases, a fact influencing the antioxidant activity. According to the “polar paradox”, hydrophilic antioxidants are more effective in nonpolar media, whereas lipophilic compounds are better antioxidants in polar media. However, several authors have reported that some compounds do not comply with the polar paradox and interpreted the behavior of phenolic compounds in emulsified systems using a different approach known as the “cutoff theory” [40,41]. Sorensen *et al*. [34] reported that caffeic acid and rutin inhibited the development of PV during the entire storage period in Citrem-stabilized emulsions at pH 6. Furthermore, the most water-soluble compound, caffeic acid, showed different effects depending on pH and emulsifier type. Thus, it was a strong pro-oxidant at pH 3 (with or without iron), but at pH 6 its effect depended on the emulsifier type and on the presence of iron. In addition Medina *et al*. [37] reported that at pH 6, caffeic acid was able to reduce the amount of peroxides formed in emulsions containing Tween, but increased the formation of volatiles. Conde *et al*. [2] found that caffeic acid (5 mmol/kg emulsion) showed good antioxidant properties in 30% sunfloweroil-in-water emulsions at pH 5.4 during storage at 50 °C. The same author [40] reported that higher concentrations of rutin and apigenin in the rafined extracts produced from chestnut burs retarded the formation of hydroperoxides in oil-in-water emulsions.

The ability of a compound to inhibit lipid oxidation could be influenced by its interactions with other antioxidants [42]. Synergy between antioxidants has been reported in a range of different media, including oils, emulsions, liposomes, microemulsions, fish and meat muscles. In some reports, the effects of antioxidants used in a combination could only be described as additive, but the term synergy should be restricted to situations where the mixture of antioxidants has a greater impact than the sum of their separate effects. Synergy between antioxidants may vary both with the medium and the nature of the lipids. Caffeic acid was effective in protecting α-tocopherol in retarding lipid oxidation in the fish muscle [43,44]. α-Tocopherol showed a strong synergistic effect with queretin in the methyl oleate in water emulsion, but the effect was reduced in phospolipd liposomes and the combination of α-tocopherol and quercetin had a shorter induction time than quercetin alone, when the oxidative stability was assessed in oil by the Rancimat test [45].

## 4. Conclusions

This study showed that the extract of *Artemisia annua* provides protection against the oxidative deterioration of oil-in-water emulsion. In addition, food emulsions appear to be useful vectors in supplying the daily dosage of *A. annua* extract in consumers, which may positively affect their health. Moreover, considering consumer’s preference for antioxidants from natural sources, these results could offer the basis for their more systematic use by food industry. Further research into the enrichment of food products with bioactive substances extracted from *A. annua* should be conducted because we still have no sufficient knowledge about their activity during food processing, or about their interactions with other food components.

## References

[1] Alamed J., Chaiyasit W., McClements D.J., Decker E.A. (2009). Relationships between free radical scavenging and antioxidant activity in foods. J. Agric. Food Chem..

[2] Conde E., Gordon M.H., Moure A., Dominguez H. (2011). Effects of caffeic acid and bovine serum albumin in reducing the rate of development of rancidity in oil-in-water and water-in-oil emulsions. Food Chem..

[3] Waraho T., Cardenia V., Nishino Y., Seneviratne K.N., Rodriguez-Estrada M.T., McClements D.J., Decker E.A. (2012). Antioxidant effects of mono- and diacylglycerols in non-stripped and stripped soybean oil-in-water emulsions. Food Res. Int..

[4] Brewer M.S. (2011). Natural antioxidants: Sources, compounds, mechanisms of action, and potential applications. Compr. Rev. Food Sci. Food Saf..

[5] Viuda-Martos M., El Gendy A.E.-N.G.S., Sendra E., Fernández-López J., Abd El Razik K., Omer E., Pérez-Alvarez J. (2010). Chemical composition and antioxidant and anti-Listeria activities of essential oils obtained from some Egyptian plants. J. Agric. Food Chem..

[6] Ferreira J.F.S., Luthria D.L., Sasaki T., Heyerick A. (2010). Flavonoids from *Artemisia annua* L. as antioxidants and their potential synergism with artemisinin against malaria and cancer. Molecules.

[7] Radulović N.S., Randjelović P.J., Stojanović N.M., Blagojević P.D., Stojanović-Radić Z.Z., Ilić I.R., Djordjević V.B. (2013). Toxic essential oils. Part II: Chemical, toxicological, pharmacological and microbiological profiles of *Artemisia annua* L. volatiles. Food Chem. Toxicol..

[8] Ćavar S., Maksimović M., Vidic D., Parić A. (2012). Chemical composition and antioxidant and antimicrobial activity of essential oil of *Artemisia annua* L. from Bosnia. Ind. Crops Prod..

[9] Huang L. (1993). Antipyretic and anti-inflammatory effects of *Artemisia annua* L.. Zhongguo Zhong Yao Za Zhi.

[10] Kim E.-K., Lee S.-J., Moon S.-H., Jeon S.-T., Ahn C.-B., Kim B., Lim B.-O., Park P.-J. (2009). Free radical scavenging activity and comparative proteomic analysis of antioxidative protein against H2O2-induced oxidative stress in neuronal cells. Food Chem..

[11] Chan H.W., Singh N.P., Lai H.C. (2013). Cytotoxicity of dihydroartemisinin toward Molt-4 cells attenuated by *N*-tert-butyl-alpha-phenylnitrone and deferoxamine. Anticancer Res..

[12] Singh N.P., Ferreira J.F., Park J.S., Lai H.C. (2011). Cytotoxicity of ethanolic extracts of *Artemisia annua* to Molt-4 human leukemia cells. Planta Med..

[13] Nibret E., Wink M. (2010). Volatile components of four Ethiopian Artemisia species extracts and their *in vitro* antitrypanosomal and cytotoxic activities. Phytomedicine.

[14] Singleton V.L., Orthofer R., Lamuela-Raventós R.M. (1999). Analysis of total phenols and other oxidation substrates and antioxidants by means of folin-ciocalteu reagent. Methods Enzymol..

[15] Zhishen J., Mengcheng T., Jianming W. (1999). The determination of flavonoid contents in mulberry and their scavenging effects on superoxide radicals. Food Chem..

[16] Almajano M.P., Carbo R., Jimenez J.A.L., Gordon M.H. (2008). Antioxidant and antimicrobial activities of tea infusions. Food Chem..

[17] Skowyra M., Falguera V., Gallego G., Peiró S., Almajano M.P. (2013). Antioxidant properties of aqueous and ethanolic extracts of tara (*Caesalpinia spinosa*) pods *in vitro* and in model food emulsions. J. Sci. Food Agric..

[18] Gallego M.G., Gordon M.H., Segovia F.J., Skowyra M., Almajano M.P. (2013). Antioxidant properties of three aromatic herbs (rosemary, thyme and lavender) in oil-in-water emulsions. J. Am. Oil Chem. Soc..

[19] Yoshida H., Kajimoto G., Emura S. (1993). Antioxidant effects of d-tocopherols at different concentrations in oils during microwave heating. J. Am. Oil Chem. Soc..

[20] Frankel E.N. (1998). Lipid Oxidation.

[21] Firestone D., American Oil Chemists’ Society (1997). AOCS Official Method Cd 8-53.

[22] Maqsood S., Benjakul S. (2010). Comparative studies of four different phenolic compounds on *in vitro* antioxidative activity and the preventive effect on lipid oxidation of fish oil emulsion and fish mince. Food Chem..

[23] Gouveia S.C., Castilho P.C. (2013). *Artemisia annua* L.: Essential oil and acetone extract composition and antioxidant capacity. Ind. Crops Prod..

[24] Iqbal S., Younas U., Chan K.W., Zia-Ul-Haq M., Ismail M. (2012). Chemical composition of *Artemisia annua* L. leaves and antioxidant potential of extracts as a function of extraction solvents. Molecules.

[25] Zheng W., Wang S.Y. (2001). Antioxidant activity and phenolic compounds in selected herbs. J. Agric. Food Chem..

[26] Carvalho I.S., Cavaco T., Brodelius M. (2011). Phenolic composition and antioxidant capacity of six artemissia species. Ind. Crops Prod..

[27] Carbonara T., Pascale R., Argentieri M.P., Papadia P., Fanizzi F.P., Villanova L., Avato P. (2012). Phytochemical analysis of a herbal tea from *Artemissia annua* L.. J. Pharm. Biomed. Anal..

[28] Ivanescu B., Vlase L., Corciova A., Lazar M.I. (2010). HPLC-DAD_MS study of polyphenols from *Artemisia abstinthium*, *A. annua* and *A. vulgaris*. Chem. Nat. Compd..

[29] Kiokias S., Dimakou C., Oreopoulou V. (2009). Activity of natural carotenoid preparations against the autoxidative deterioration of sunflower oil-in-water emulsions. Food Chem..

[30] Ramful Aumjaud D.B., Neergheen V.S., Soobrattee M., Googoolye K., Aruoma O.I., Bahorun T. (2011). Polyphenolic content and antioxidant activity of *Eugenia pollicina* leaf extract *in vitro* and in model emulsion systems. Food Res. Int..

[31] Roedig-Penman A., Gordon M. (1997). Antioxidant properties of catechins and green tea extracts in model food emulsions. J. Agric. Food Chem..

[32] Decker E.A., Warner K., Richards M.P., Shahidi F. (2005). Measuring antioxidant effectiveness in food. J. Agric. Food Chem..

[33] Sun Y.-E., Wang W.-D., Chen H.-W., Li C. (2011). Autoxidation of unsaturated lipids in food emulsion. Crit. Rev. Food Sci. Nutr..

[34] Sørensen A.-D.M., Haahr A.-M., Becker E.M., Skibsted L.H., Bergenståhl B., Nilsson L., Jacobsen C. (2008). Interactions between iron, phenolic compounds, emulsifiers, and pH in omega-3-enriched oil-in-water emulsions. J. Agric. Food Chem..

[35] García-Iñiguez de Ciriano M., Rehecho S., Calvo M.I., Cavero R.Y., Navarro I., Astiasarán I., Ansorena D. (2010). Effect of lyophilized water extracts of Melissa officinalis on the stability of algae and linseed oil-in-water emulsion to be used as a functional ingredient in meat products. Meat Sci..

[36] Dimakou C., Oreopoulou V. (2012). Antioxidant activity of carotenoids against the oxidative destabilization of sunflower oil-in-water emulsions. LWT-Food Sci. Technol..

[37] Medina I., Undeland I., Larsson K., Storrø I., Rustad T., Jacobsen C., Gallardo J.M. (2012). Activity of caffeic acid in different fish lipid matrices: A review. Food Chem..

[38] Almajano M.P., Carbo R., Delgado M.E., Gordon M.H. (2007). Effect of pH on the antimicrobial activity and oxidative stability of oil-in-water emulsions containing caffeic acid. J. Food Sci..

[39] Zhou L., Elias R.J. (2013). Antioxidant and pro-oxidant activity of (−)-epigallocatechin-3-gallate in food emulsions: Influence of pH and phenolic concentration. Food Chem..

[40] Conde E., Moure A., Domínguez H., Gordon M.H., Parajó J.C. (2011). Purified phenolics from hydrothermal treatments of biomass: Ability to protect sunflower bulk oil and model food emulsions from oxidation. J. Agric. Food Chem..

[41] Poyato C., Navarro-Blasco I., Calvo M.I., Cavero R.Y., Astiasaran I., Ansorena D. (2013). Oxidative stability of O/W and W/O/W emulsions: Effect of lipid composition and antioxidant polarity. Food Res. Int..

[42] Alamed J., Chaiyasit W., McClements D.J., Decker E.A. (2009). Relationships between free radical scavenging and antioxidant activity in foods. J. Agric. Food Chem..

[43] Gordon M.H., Decker E.A., Elias R.J., McClements D.J. (2010). Effects of Food Structure and Ingredient Interactions on Antioxidant Capacity. Oxidation in Foods and Beverages and Antioxidant Applications: Understanding Mechanisms of Oxidation and Antioxidant Activity.

[44] Iglesias J., Pazos M., Andersen M.L., Skibsted L.H., Medina J. (2009). Caffeic acid as antioxidant in fish muscle: Mechanism of synergism with endogenous ascorbic acid and α-tocopherol. J. Agric. Food Chem..

[45] Becker E.M., Ntouma G., Skibsted L.H. (2007). Synergism and antagonism between quercetin and other chain-breaking antioxidants in lipid systems of increasing structural organisation. Food Chem..

